# Treatment of an Opposing Metabolic Situation: GLUT1‐Deficiency Syndrome and Type 1 Diabetes

**DOI:** 10.1002/jmd2.70007

**Published:** 2025-03-25

**Authors:** Anna K. Schoenlaub, Alexander Hoeller, Sabine Hofer, Edda Haberlandt, Elisabeth Steichen‐Gersdorf, Daniela Karall, Dorottya Forster, Sabine Scholl‐Bürgi

**Affiliations:** ^1^ Department of Pediatrics I Medical University of Innsbruck, Tyrolean State Hospitals Innsbruck Austria; ^2^ Division of Nutrition and Dietetics University Hospital Innsbruck Innsbruck Austria; ^3^ Institute of Public Health, Medical Decision Making and Health Technology Assessment, Department of Public Health, Health Services Research and Health Technology Assessment UMIT TIROL‐University for Health Sciences and Technology Hall in Tirol Austria; ^4^ Digital Health Information Systems, Center for Health & Bioresources AIT Austrian Institute of Technology Graz Austria; ^5^ Department of Pediatrics Hospital of Dornbirn Dornbirn Austria

**Keywords:** glucose transporter type 1 deficiency syndrome, insulin therapy, ketoacidosis, ketogenic diet therapy, modified Atkins diet, type 1 diabetes

## Abstract

Glucose transporter type 1 deficiency syndrome (GLUT1‐DS) is a rare inborn disorder of metabolism leading to encephalopathy due to disturbed glucose transport via the blood–brain‐barrier and consecutive energy deficit of the brain. Since ketone bodies can serve as an alternative fuel for the brain, ketogenic diet therapies (KDT) are the treatment of choice for these patients. KDT refers to all forms of nutrition that lead to the formation of ketone bodies. We describe a 15‐year‐old girl with GLUT1‐DS who was effectively treated with a form of KDT, a modified Atkins diet (MAD), and developed type 1 diabetes. After correction of the initial diabetic ketoacidosis (DKA), insulin pump treatment was started while staying on MAD. With this treatment regimen, no further DKA episodes occurred within 2 years of follow‐up, current HbA1c 6.9%. Treatment of GLUT1‐DS by KDT and type 1 diabetes (T1D) by insulin at the same time is challenging but feasible. The initial manifestation phase of T1D is critical and is made even more difficult by an already performed KDT. Target ranges for blood glucose AND β‐hydroxybutyrate levels must be defined to optimize the insulin dosage. Additionally, patients, families, and caregivers need to be aware of the risk of this particular metabolic situation.

## Graphical abstract

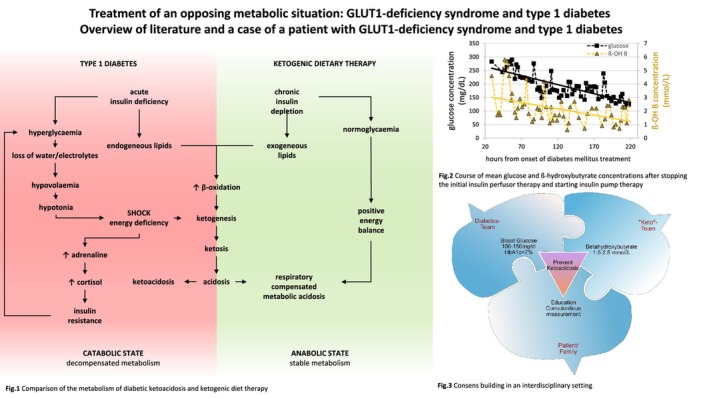

1

**Summary**

- The simultaneous treatment of GLUT1‐deficiency syndrome by ketogenic diet therapy and type 1 diabetes by insulin can be managed successfully.

## 2 Introduction

Glucose transporter type 1 deficiency syndrome (GLUT1‐DS) is a rare inborn disorder of metabolism that leads to encephalopathy due to disturbed glucose transport via the blood–brain‐barrier and consecutive energy deficit of the brain. The underlying condition is a pathogenic variant of the *SLC2A1* gene. Symptoms include early‐onset epilepsy, complex movement disorders, and developmental delay. Patients typically present during the first years of life with eye‐head movement abnormalities and generalized seizures, also, ataxia or unsteady gait may occur, and all symptoms are age‐specific [1, 2, 3]. Deficiency of glucose transporter type 1 (GLUT1) leads to hypoglycorrhachia, which means an impaired glucose concentration in cerebrospinal fluid (CSF) [4]. Diagnosis can be confirmed by characteristic clinical symptoms, detection of hypoglycorrhachia, and identification of a pathogenic *SLC2A1*‐variant in molecular genetic analysis [1, 5]. The first‐line therapy is a ketogenic diet therapy (KDT), a high‐fat, low‐carbohydrate, and age‐adequate‐protein diet [1, 3]. KDT provides ketone bodies as an alternative energy source that can cross the blood–brain barrier bypassing GLUT1 and with the help of the monocarboxylate transporter type 1 [2, 6]. Ketone bodies are generated from fatty acids by the liver. Classic ketogenic diet (cKD) and modified Atkins Diet (MAD) are common forms of KDT [7, 8, 9]. The cKD is based on strict proportion from fat to carbohydrates and protein together. In contrast, the MAD requires a fixed amount of carbohydrates per day. For patients with GLUT1‐DS it is recommended to continue the KDT at least into early adulthood [1], as the brain has an increased demand of energy while development [2].

KDT is also a therapy for refractory epilepsies [10, 11]. Additionally, KDT is discussed as add‐on strategy for type 2 diabetes as it reduces the need for insulin. However, there is no evidence that patients with type 1 diabetes (T1D) should be treated with KDT [12]. We are aware of five case reports describing the challenges of KDT and type 1 diabetes in children [13, 14, 15, 16, 17] and report on a 15‐year‐old girl with GLUT1‐DS performing MAD and presenting with ketoacidosis due to type 1 diabetes onset.

## 3 Case Presentation

A 15‐year‐old girl with a GLUT1‐DS on MAD presented with weight loss, abdominal pain, and high ketosis.

The past medical history was remarkable for eye‐head movement abnormalities at the age of 4 weeks, delayed development of head control, absence of crawling, and delayed atactic gait. At the age of 10 months, seizures occurred, and they turned out to be resistant to antiseizure medication. At the age of 10 years, hypoglycorrhachia (45 mg/dL) was detected, and the CSF to blood glucose ratio was reduced (0.42) [18]. A molecular genetic analysis showed a de novo heterozygous missense mutation of the *SLC2A1* gene (p.Val391Leu). MAD was initiated (12 g of carbohydrates per day) at the age of 10 years, and medium‐chain triglycerides (MCT) were added twice a day. With this approach, the patient became seizure‐free, and her gait improved. Her cognitive development improved.

At the age of 15 years, she presented during a routine control with 4 kg of weight loss (weight 10th percentile, body height 90th percentile) and abdominal pain. Typical signs for T1D, such as polydipsia and polyuria were missing. The initial blood gas analyses showed elevated blood glucose concentration (301 mg/dL) and ketoacidosis (pH 7.16; serum bicarbonate 10.2 mmol/L, base excess −19.5 mmol/L, pCO_2_ 22 mmHg), the urine sample showed glucosuria and ketonuria. Further laboratory findings completed the diagnosis of T1D with elevated HbA1c of 9.6% (81 mmol/mol) and low C‐peptide (1.2 μg/L) levels, insulin level was low (1.7 mU/L). T1D‐specific antibodies (GAD antibodies, IA2‐antibodies) were not detected at the time.

**FIGURE 1:**
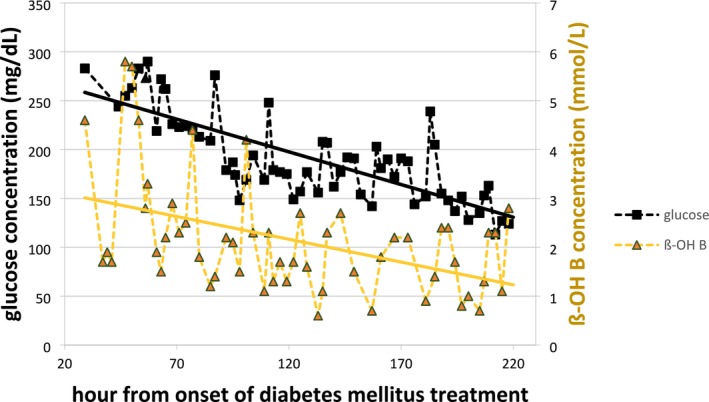
Course of mean glucose and ß‐hydroxybutyrate concentrations after stopping the initial insulin perfusor therapy and starting insulin pump therapy.

Rehydration was performed according to the DKA protocol (ISPAD Guidelines) with physiologic saline, glucose infusion was added when glucose concentration was 183 mg/dL (hour 5–16: 2.5–5 g/h; hour 17–40: 1.25 g/h). In addition, rapid acting insulin was applied by syringe driver (minimal 0.025 IU/kg h; maximal 0.034 IU/kg h). The patient still received a restricted amount of carbohydrates orally as on MAD (12 g carbohydrate orally per day). During this acute phase (first 72 h) of DKA treatment, β‐hydroxybutyrate concentrations stayed between 1.2 and 5.6 mmol/L while mean glucose concentrations decreased to physiologic levels and acidosis improved (Figure 1).

**FIGURE 2:**
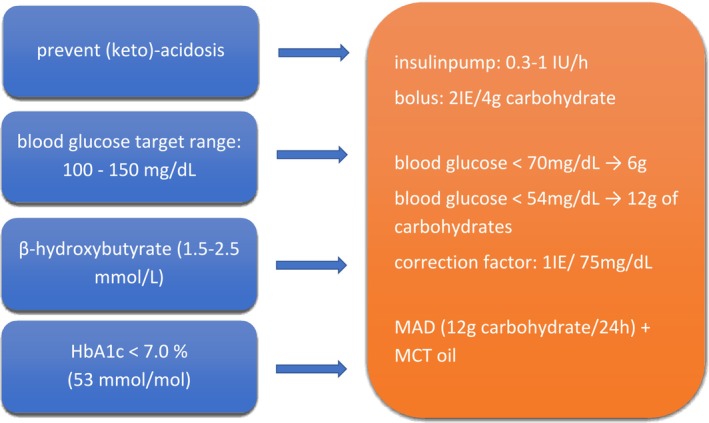
Interdisciplinary consensus on how to prevent ketoacidosis and keep ketosis.

In an interdisciplinary consensus of pediatric diabetologists, specialists for metabolic diseases, and pediatric neurologists, the following goals were defined (Figure 2):

- immediate and sufficient treatment of DKA,
- blood glucose target between 100 and 150 mg/dL to prevent hypoglycaemia,
- moderate ketosis should be maintained to avoid seizures and movement disorders (range between 1.5 and 2.5 mmol/L β‐hydroxybutyrate), and
- long term metabolic control HbA1c < 7.0% (53 mmol/mol).

A continuous subcutaneous insulin pump using rapid‐acting insulin analogues was initiated at the third day after the diagnosis of T1D 0.3 IE/h of insulin was given initially and gradually increased to 1 IE/h. The correction factor was 1 IE of insulin per 75 mg/dL when glucose concentrations reached values above 150 mg/dL. For blood glucose concentrations below 70 mg/dL (or 54 mg/dL), 6 g (or 12 g) of carbohydrates, were administered. Among these, ketone levels decreased to 0.7 and 0.9 mmol/L in the morning, leading to dizziness. Therefore, 0.1 mL/kg MCT oil was added in the evening, and the base rate of insulin at night was reduced to 0.8 IU/h to keep ketones in the target range in the morning.

No episode of DKA occurred over 24 months of follow‐up with insulin pump treatment. HbA1c is 6.9% (52 mmol/mol), and the current insulin dosage is 0.6 IE/kg body weight per day. β‐Hydroxybutyrate levels vary between 0.4 and 2.2 mmol/L. The patient did not show any seizures or paroxysmal movement disorders within an observation period of 2 years.

## 4 Discussion and Overview of Literature

Our report summarizes the management of a girl with GLUT1‐DS on KDT diagnosed with T1D. Patients with T1D mainly present with ketoacidosis, which reflects a lack of insulin. In contrast, patients on KDT are known to have ketosis (not ketoacidosis) with adequate low insulin concentrations. Ketosis is essential for patients with GLUT1‐DS as ketone bodies provide a sufficient energy supply for the brain. Therefore, ketosis but not ketoacidosis is a requested state in these patients. As insulin inhibits ketogenesis, the therapy of insulin deficiency in T1D must be adapted to the specific condition in the context of KDT. In our patient, this meant that the insulin dose was lower than in other T1D patients and that moderate β‐hydroxybutyrate elevations were not indicative of DKT.

GLUT1 is a passive transporter. It could therefore be assumed that glucose transport into the brain increases when the glucose concentrations in the blood are higher, as is the case with T1D. But the exact affinity and diffusion capacity of GLUT1 in different *SLC2A1* variants are not known. For this reason, it seems risky to consider the higher glucose levels in T1D as sufficient for the constant energy supply of the brain (in GUT1‐DS) especially since the goal of T1D therapy is to achieve normoglycaemia.

As the initial manifestation phase of T1D is particularly critical and is made even more difficult by KDT, this case report focuses on the initial phase of T1D onset but also describes the clinical course over the first 2 years of T1D. To the best of our knowledge, this is the first patient with GLUT1‐DS under KDT in whom T1D has been described. Additionally, a correlation between GLUT1‐DS and T1D is not yet known.

There are two fundamentally different situations: on the one hand, there can be an initial manifestation of a T1D under KDT, and on the other hand, a KDT can be started despite an underlying T1D. In both cases, the start of additional therapy for the biochemically contradictory situation is critical. Unfortunately, there is no comprehensive literature or guidelines for either.

*Onset of T1D in patients with KDT* (Table 1a): Henwood et al. [13] reported a girl with pyruvate dehydrogenase deficiency (PDH deficiency). She started KDT at the age of 2 years and 9 months. At the age of 4 years, she presented with ketoacidosis and T1D was diagnosed. She also received insulin and KDT without complications, especially no further ketoacidosis. Aguirre Casteneda et al. [14] published a case of a girl who started with KDT at the age of 10 months because of intractable childhood epilepsy and showed significant improvement. She was 2 years old at the onset of T1D. She was successfully treated with combined KDT and insulin for 10 months. KDT was stopped because the diet was difficult to adhere.

**TABLE 1:** Review of literature and comparison of case reports on the KDT and therapy for T1D.

		Diagnosis	Age at start of KDT	Type of KDT	Age at onset of type 1 diabetes	Treatment for type 1 diabetes	Outcomes
(a) Onset of T1D in patients with KDT	Henwood et al. [13]	Pyruvate dehydrogenase deficiency	2a 9 months	cKDT	4a	Glargine insulin 0.3 U/kg at bedtime, after 6 months 0.53 U/kg in two doses	*Admission*: Glucose 575 mg/dL, HbA1c: 6.9%, urine ketone: “large,” serum lactate: 2.1 mmol/L
						Lispro insulin bolus before meals for glucose > 250 mg/dL or B‐OHB > 2.5 mmol/L	*10 months*: B‐OHB: 0.7–2 mmol/L, HbA1c: 5.1%
							*28 months*: No hypoglycaemic events, no diabetic ketoacidosis
	Aguirre Castenada et al. [14]	Intractable childhood epilepsy	10 months	cKDT 4:1	2a	Glucose range (fasting): 100–150 mg/dL	*Admission*: Random glucose: 362 mg/dL, HbA1c: 7.3%, B‐OHB: 7.7 mmol/L
						Ketonuria goal: moderate	*6 months*: Random glucose: 179 mg/dL, HbA1c: 6.8%, B‐OHB: 2.2 mmol/L
						Glargine insulin 0.3 U/kg at bedtime (adjusted to keep glucose within the goal range)	*10 months (KDT stopped)*: Random glucose: 274 mg/dL, HbA1c: 7.2%, B‐OHB: n.a.
						Asparte insulin with or without large ketonuria before meals for glucose 200 mg/dL	*22 months (KDT stopped)*: Random glucose: 300 mg/dL, HbA1c: 8.4%, B‐OHB: n.a.
(b) Initiation of KDT in patients with T1D	Dressler et al. [15]	Right‐sided hemiparesis, and focal epilepsy due to a malformation of cortical development	3a 6 months	cKDT 3:1–2.5:1 (strong ketosis)	18 months	Continuous subcutaneous insulin infusion	*6 months*: Plasma glucose: 140 mg/dL, HbA1c: 6.7%, B‐OHB: n.a., Urine ketone: 3+
							*10 months*: Plasma glucose: 98 mg/dL, HbA1c: 6.8%, B‐OHB: n.a., Urine ketone: 3+
							*13 months*: Plasma glucose: 192 mg/dL, HbA1c: 6.2%, B‐OHB: n.a., Urine ketone: 3+, No hypoglycaemia, no ketoacidosis
	Aylward et al. [16]	Myoclonic astatic epilepsy	4a 11 months	cKDT 4:1	3.8a	Glucose range 72–180 mg/dL	*2 months*: B‐OHB: 2.3–5.3 mmol/L
						Blood ketones > 4 mmol/L	*6a*: HbA1c: 5.7%–6.4%, no hypoglycaemia, no ketoacidosis
						Basal (glargine insulin) bolus (lispro insulin) regime	
	Zegarra et al. [17]	Microcephaly, epilepsy, and diabetes syndrome (MEDS)	9 months	cKDT 3:1	4 months	0.01 U/kg d	

Abbreviations: B‐OHB, β‐hydroxybutyrate; cKD, classic ketogenic diet; DKA, diabetic ketoacidosis; KDT, ketogenic diet therapy.

*Initiation of KDT in patients with T1D* (Table 1b): Patients with T1D have a 2‐to‐6‐fold higher risk of epilepsy [19] and may benefit from KDT when the epilepsy is refractory. There are not many reports of patients with T1D who have nevertheless started KDT. Dressler et al. [15] reported a female patient who was 18 months at the onset of T1D. Due to a cortical malformation, she suffered from a focal epilepsy; therefore, she started KDT at the age of 3.5 years. She became seizure‐free. KDT was stopped after 15 months because the patient refused ketogenic meals. Aylward et al. [16] published a case report about a boy with T1D at the age of 3.8 years who showed first seizures 1 week after initiation of insulin therapy. During the course, myoclonic astatic epilepsy was diagnosed with refractory seizures. One year later, a KDT was initiated and administered by gastrostomy tube and seizures frequency decreased significantly. The patient received a basis bolus regime of insulin and over the following 6 years no episodes of severe hypoglycaemia or DKA occurred. Furthermore, his cognition improved. Most recently, Zegarra et al. [17] report about an infant with microcephaly, epilepsy, and diabetes syndrome (MEDS). Seizures began at the age of 2 months and turned out to be refractory to medication. At the age of 4.5 months, insulin therapy was started because of T1D. 4.5 months later, a KDT (cKD [3:1]) was initiated due to infantile spasms. Initially, insulin was decreased to 0.01 units/kg/day not to inhibit ketone production. Two months later, the patient developed a DKA due to a viral infection with diarrhea and vomiting. She needed intravenous fluid, insulin, and dextrose. Afterwards, KDT was restarted as it was effective in epilepsy. But as the next viral infection led to DKA, the KDT was weaned.

The development of a DKA is reported in the most recent case [17], where early signs of DKA led to delayed treatment due to the long distance to the hospital. The risk of masked metabolic decompensation must be explained to the patient and the patient's family. They must learn about the pathophysiology of T1D and the reciprocal effect to the KDT.

In summary, a combination of these two metabolic conditions is a balancing act, but our case report may be helpful for these patients, showing that combining both therapies is possible.

## Author Contributions

Anna K. Schoenlaub researched and summarized clinical data, researched literature, and wrote the first draft of the manuscript. Alexander Hoeller researched and summarized clinical data, researched literature, contributed to discussion and reviewed and edited the manuscript. Sabine Hofer contributed to discussion and reviewed and edited the manuscript. Edda Haberlandt contributed to discussion and reviewed and edited the manuscript. Elisabeth Steichen‐Gersdorf contributed to discussion and reviewed and edited the manuscript. Daniela Karall contributed to discussion and reviewed and edited the manuscript. Dorottya Forster contributed to discussion and reviewed and edited the manuscript. Sabine Scholl‐Bürgi researched and summarized clinical data, contributed to discussion and reviewed and edited the manuscript. Sabine Scholl‐Bürgi serves as guarantor for the article, accepts full responsibility for the work, had access to the data, and controlled the decision to publish. All authors approved the final version of the manuscript.

## Ethics Statement

The authors have nothing to report.

## Consent

A patient's consent statement is available upon request.

## Conflicts of Interest

The authors declare no conflicts of interest.

## Data Availability

The data that support the findings of this study are available on request from the corresponding author. The data are not publicly available due to privacy.
